# Construction of ZnO/r-GO Composite Photocatalyst for Improved Photodegradation of Organic Pollutants

**DOI:** 10.3390/molecules30051008

**Published:** 2025-02-21

**Authors:** Yun Ding, Wenzhen Qin, Huihua Zhu, Yuhua Dai, Xiaowei Hong, Suqin Han, Yu Xie

**Affiliations:** 1 School of Environmental and Chemical Engineering, Nanchang Hangkong University, Nanchang 330063, China; yunding@163.com (Y.D.); qwz1417@163.com (W.Q.); huihuazhu@163.com (H.Z.); dyh@nchu.edu.cn (Y.D.); 2 Yangxin County Special Education School, Binzhou 310000, China; 15065265752@163.com

**Keywords:** zinc oxide, graphene oxide, photocatalytic activity, simulated sunlight irradiation

## Abstract

In this work, a simple hydrothermal method was used to prepare a series of ZnO/r-GO (ZGO-x) catalysts. The obtained products were subjected to a series of characterizations, which showed that the zinc oxide particles were deposited onto r-GO and that the crystal structure was not disrupted. In addition, due to the large specific surface area and the good electrical conductivity of r-GO, more photogenerated electrons can be rapidly transferred from ZnO to r-GO to participate in the reaction, thus improving the photocatalytic performance. The degradation rate of the ZGO-3 sample reached 100% for RhB after simulated sunlight irradiation for 150 min, whereas the pure ZnO degraded RhB by about 83% under the same environment. ZGO-3 also showed the best photocatalytic degradation of methyl orange, with 100% degradation in 180 min, whereas pure ZnO degraded only 87.64% of methyl orange under solar irradiation.

## 1. Introduction

In recent years, with the rapid development of industry, the discharge of organic pollutants has been increasing, which has brought great harm to animals, plants, and human society. ZnO nanoparticles have attracted much attention in terms of the photodegradation of organic pollutants due to their excellent photovoltaic properties, good thermal stability, and low cost, etc. [1,2,3,4,5]. However, the photocatalytic activity of ZnO is limited due to the easy complexation of photogenerated electron–hole pairs and the narrow spectral absorption range [6]. Therefore, the inhibition of photogenerated electron–hole pair complexation and enhancement in visible light utilization are the main ways of improving photocatalytic performance [7].

Two-dimensional carbon nanomaterials such as graphene oxide (GO) and reduced graphene oxide (r-GO) exhibit excellent electrical conductivity, a large surface area, strong light absorption, and efficient electron transfer capabilities, thus garnering a dedicated following in the photocatalysis field [8,9]. Significantly, the surface of r-GO is enriched with a variety of oxygen-containing functional groups, which is conducive to the transfer of electron holes [10,11]. For example, Liu et al. constructed a r-GO/TP-COF heterostructure photocatalyst to improve photocatalytic properties [12]. Therefore, r-GO can be employed as a charge movement accelerator to additionally enhance photocatalytic properties. To date, ZnO/r-GO heterostructure photocatalysts have been constructed using the following three methods: (1) the reduction of graphene on the basis of the structure of ZnO [13], (2) the preparation of ZnO on r-GO [14], and (3) the preparation of ZnO along with graphene reduction [15]. The previous two methods require the optimization of ZnO or GO. Therefore, the preparation of ZnO along with graphene reduction is considered as an effective way to fabricate ZnO/r-GO nanocomposites [16,17,18]. However, the agglomeration of ZnO on r-GO limits the electron mobility of the catalyst during photocatalysis [19]. Therefore, how to evenly distribute ZnO nanoparticles on r-GO has become the focus of current research [20,21].

In this work, different ratios of ZnO nanoparticles were homogeneously dispersed on the surface of r-GO to design a ZnO/r-GO heterostructure hydrothermally. The reasons for the improved photocatalytic performance were investigated using ultraviolet–visible diffuse reflectance spectroscopy (UV-vis), the Brunauer–Emmett–Teller (BET) method, photocurrent, and so on. The ZnO/r-GO photocatalyst displays a remarkably raised RhB degradation rate compared to the pure ZnO nanoparticles and possesses good stability in repeated catalytic experiments.

## 2. Results and Discussion

### 2.1. Structure and Physical Properties

**Figure 1:**
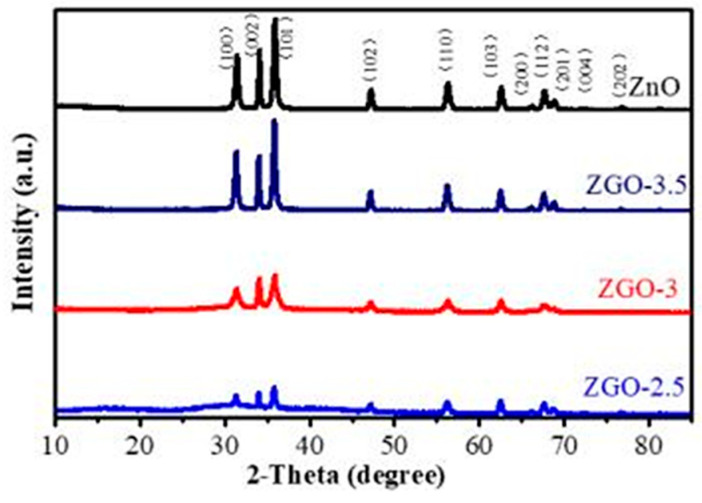
XRD patterns of series of ZGO-x catalyst samples.

Figure 1 shows the XRD spectra of ZnO, ZGO-2.5, ZGO-3, and ZGO-3.5, which were used to determine the crystalline state of the Zn species in the materials. All the diffraction peaks conform to the standard data for the fibrillated zincite structure (JPCDS 36-1451), which suggest that the presence of r-GO does not lead to the emergence of a new crystalline form of ZnO [22]. Moreover, the typical diffraction peaks of r-GO at 26°(002) and 44.5°(100) are not observed in ZGO-x series catalysts, possibly due to the low r-GO content [23]. In addition, it is noticed that the intensity of the ZnO peaks increase with the increase in ZnO content, but the positions of the diffraction peaks are not shifted and no other characteristic diffraction peaks appear, indicating that the series of ZGO-x catalysts have been successfully prepared by this method.

**Figure 2:**
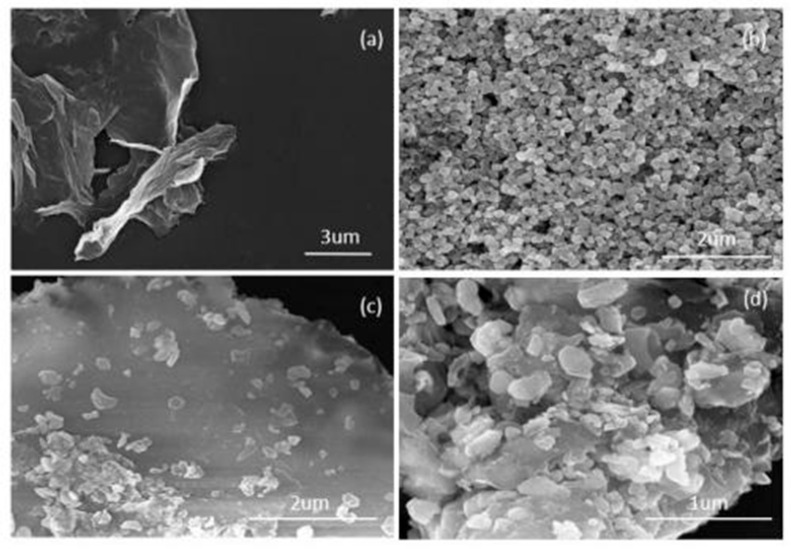
SEM images of various catalysts: (**a**) r-GO, (**b**) ZnO, (**c**) ZGO-3, (**d**) ZGO-2.5.

The micromorphology and variation in ZGO-x series samples were studied by scanning electron microscopy, as shown in Figure 2. The results show that r-GO has a two-dimensional nanosheet structure that is slightly wrinkled at the end and can be judged as having a relatively thin sheet structure [24]. According to the literature, graphene oxide consists of large continuous films with a height of 14 nanometers, which corresponds to 8–10 layers of graphene oxide. Figure 2b shows the SEM images of ZnO, which demonstrate that the prepared ZnO nanoparticles are relatively regular and homogeneous. Firstly, the nanoparticle structure of ZnO and the two-dimensional ultrathin nanosheet structure of r-GO can be clearly observed from the SEM images, and their respective morphologies remain relatively intact, indicating that ZnO and GO are not destroyed. Secondly, as the Zn content increases, the ZnO particles formed on the surface of r-GO become larger (Figure 2c,d). However, an agglomeration phenomenon occurs when excessive ZnO nanoparticles are attached onto r-GO [25]. This phenomenon reduces the light exposure area of the catalyst, which is not conducive to the generation of photogenerated electrons. More importantly, it will affect the photocatalytic cracking performance of the catalyst.

**Figure 3:**
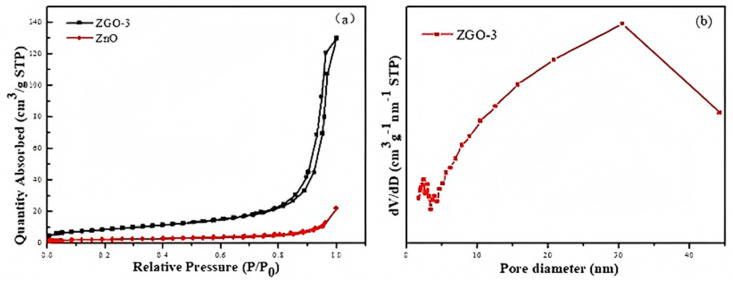
N_2_ adsorption–desorption analysis (**a**) and pore diameter distribution (**b**) of series ZGO-x samples.

Another main factor that influences the photocatalytic performance of photocatalyst is the specific surface area and pore size distribution of the catalyst [26]. The results of the adsorption–desorption isothermal curves and porous distributions of ZnO and ZGO-3 are shown in Figure 3. All samples exhibit type IV curves, which are in accordance with the results of mesoporous materials. It is also found that the positions of the H3-type rings are at a higher relative pressure (0.8–1.0), indicating the existence of large pores in the materials. This also indicates that the pores in the materials are mainly formed by flakes or particles, which coincide with the structure of r-GO flakes. The specific surface areas and pore volumes of ZnO and ZGO-3 are presented in Table 1. The specific surface area of ZGO-3 is 31.5425 m^2^/g, which is more than that of ZnO (8.1486 m^2^/g), resulting in the adsorption capacity of ZGO-3 being stronger than that of ZnO and GO. Therefore, ZGO-3 possesses more reactive sites on its surface in the catalytic reaction [27]. Meanwhile, the pore diameter of ZGO-3 is mainly distributed at about 33 nm (Figure 3b).

**Figure 4:**
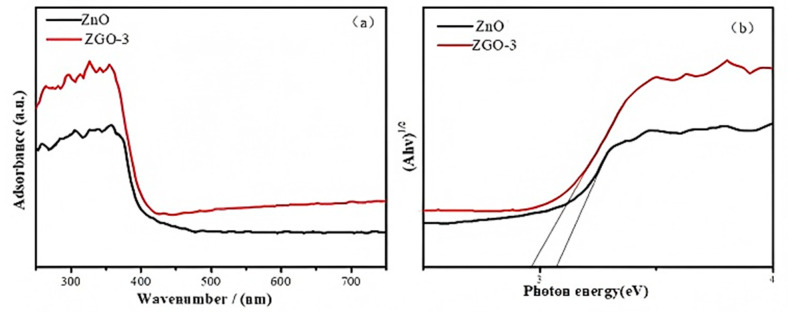
Diffuse UV-vis spectra (**a**) and band gap (**b**) of ZnO and ZGO-3.

Subsequently, to further determine the magnitude of the band gap energy and the light absorption performance of the prepared materials, DRUV-vis spectra (Figure 4) were utilized to check the ZnO and ZGO-3 samples at the ultraviolet band. The figure shows that the absorption edges of ZnO and ZGO-3 are 412.32 nm and 428.52 nm, respectively. The band gap values of ZnO and ZGO-3 are calculated to be 3.172 eV and 2.953 eV by the Kubelka–Munk equation, respectively [28]. The results show that the loading of ZnO on r-GO leads to a significant red-shift phenomenon of the catalyst. This is due to the fact that the loading of ZnO onto the r-GO surface increases the specific surface area of ZGO-3, leading to the creation of more effective absorption sites on the surface. It has been reported that the energy band structure of ZnO/GO heterojunction is similar to the simple superposition of the energy band structures of ZnO and GO. However, in the energy band structure of the ZnO/GO heterojunction, the bottom of the conduction band and the top of the valence band of ZnO are shifted down to a certain extent, and a band gap is created, which results in a high absorption coefficient in the visible range. Similarly, Geng et al. also investigated density functional theory and found that graphene synthesized on bulk ZnO exhibit a significant electronic doping phenomenon and enhancement in the work function. Taken together, this evidence can support the improved photocatalytic performance [29].

**Figure 5:**
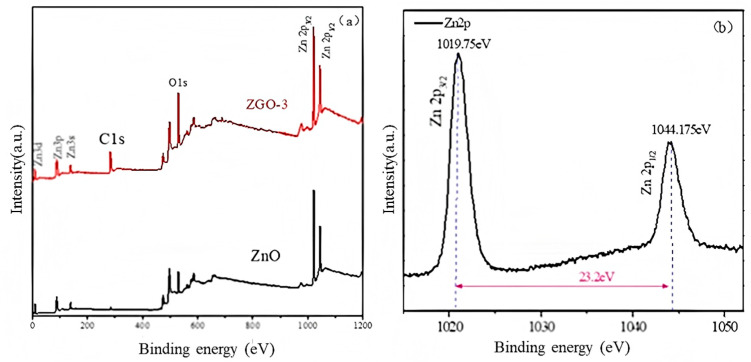
XPS spectra of full survey (**a**) and Zn2p (**b**).

To ultimately identify the valence state of the major elements in the sample, we analyzed ZnO and ZGO-3 using the XPS technique. The results of the full survey (Figure 5a) and the fine spectrum of Zn (Figure 5b) are displayed. The surface of ZnO/r-GO composites is mainly composed of C, Zn, and O elements. Moreover, the content of C in ZGO-3 is obviously more than that of the ZnO nanoparticles alone. This may be caused by the fact that a portion of r-GO in the ZGO-3 sample is reduced to r-GO. For the Zn2p spectrum of ZGO-3, the binding energy of Zn2p_1/2_ and Zn2p_3/2_ are located at 1044.175 eV and 1019.75 eV, respectively, while the spin–orbit splitting value of these peaks is 23.2 eV, which clearly indicates the presence of Zn^2+^ in ZnO [30,31,32,33].

### 2.2. Catalytic Analysis

**Figure 6:**
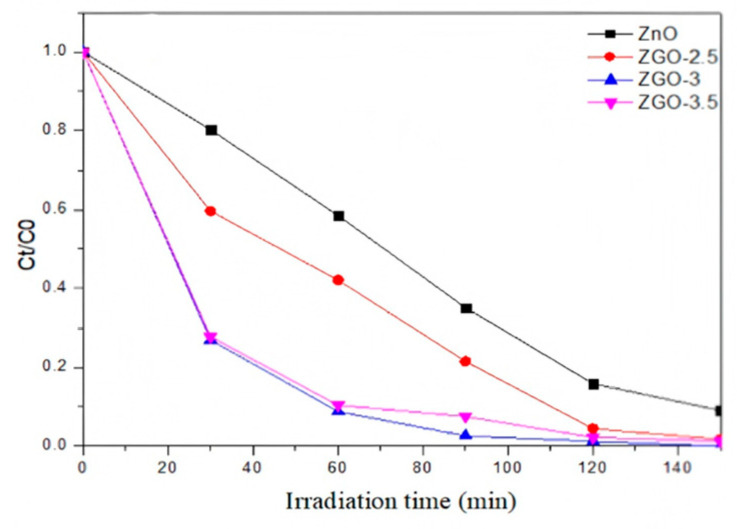
Catalytic degradation course of rhodamine B using a series of ZGO-x catalyst samples displayed under simulated sunlight.

We simulated the photodegradation of rhodamine B(RhB) to determine the photocatalytic performance of ZGO-x (Figure 6). Prior to photodegradation, we evaluated the photocatalytic performance of the r-GO sample in RhB, which showed weak photocatalytic activity under sunlight. The reaction process of RhB degradation for ZGO-x in a different time course can be discerned. The results show that the photocatalytic activity mainly comes from Zn species. Secondly, under the same conditions, the photocatalytic performance of ZGO-x series catalysts varies with the irradiation time, which should be related to the different physicochemical properties of ZGO-x. It is noteworthy that the photocatalytic activity shows an interesting trend with increasing ZnO loading in r-GO. The catalytic performance increases from ZGO-2.5 to ZGO-3, while the catalytic activity shows a decreasing trend when the ZnO loading is further raised to 3.5%. Among all the samples, the ZGO-3 sample shows the best photocatalytic performance. The degradation of rhodamine B by ZGO-3 reaches 75.2% in the first 30 min, while the degradation of r-GO is only 28.7% and that of ZnO is only 19.8%. When the illumination time is extended to 150 min, the photocatalytic performance of ZGO-3 is the best, which is an almost 100% degradation of RhB. However, ZnO degraded about 83% of RhB, and r-GO degraded about 90% of RhB.

**Figure 7:**
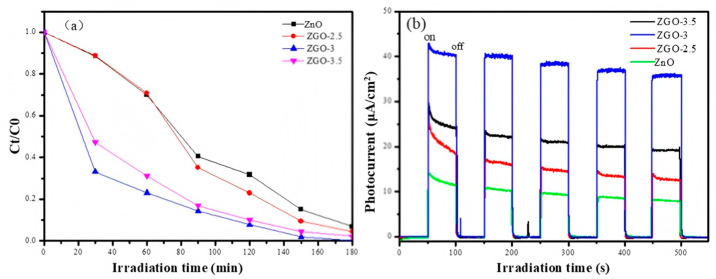
Catalytic degradation course of methyl orange using a series of ZGO-x (**a**); photocurrent test of those involved comparative catalysts (**b**).

Next, methyl orange was also chosen as the target simulated pollutant, and the photocatalytic degradation of methyl orange was conducted using the four samples above. The ZGO-3 sample shows the best photocatalytic degradation of methyl orange under simulated sunlight, with 100% degradation within 180 min, while the pure ZnO only degraded 87.64% of methyl orange under sunlight (Figure 7a). Combined with the previously described characterization, we suggest that ZnO may form a certain degree of agglomeration on the catalyst surface when excessive ZnO particles appear on the catalyst surface. In addition, the excessive introduction of the Zn element in r-GO will obviously damage the porous structure and lead to the degradation of physical structural properties. Additionally, the resulting light-exposed area of the catalyst will be reduced, which is unfavorable for the generation of photogenerated electrons.

The ability to separate electrons (e^−^) and holes (h^+^) generated by photoexcitation is also a major factor affecting photocatalytic reactions, and a photocurrent test was used to investigate the separation efficiency of the photogenerated electrons and holes of the prepared samples. In the absence of light exposure, no photocurrent is generated, whereas in the presence of light, a transient current is generated. The phenomenon suggests that when a light source irradiates a photocatalyst, directionally moving electrons are generated to form a photocurrent. As shown in Figure 7b, the photocurrent signal of the ZGO-3 sample is the strongest with a maximum of 43.5 μA/cm^2^ after five light-on/light-off cycles, which is 2.94 times higher than that of ZnO (14.8 μA/cm^2^). The results clearly indicate that the effective complexation of ZnO with r-GO results in the transfer of photoexcited electrons from ZnO to r-GO, which serves to separate photogenerated electron–hole pairs and improve the photocatalytic performance.

**Figure 8:**
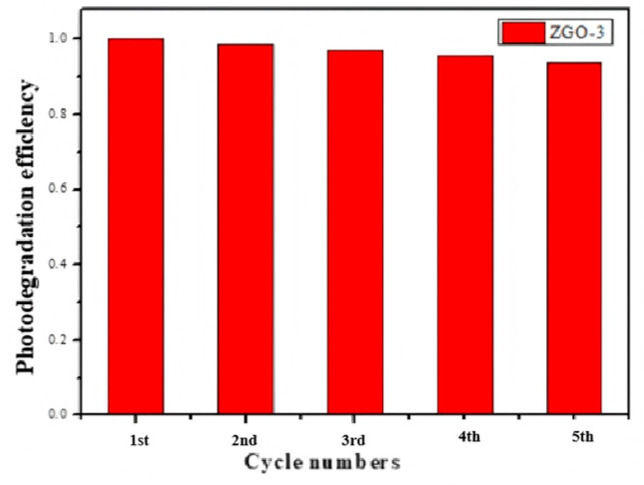
The recycling test results of ZGO-3.

We then assessed for photocatalytic stability by recovering the ZGO-3 catalyst, and the results are shown in Figure 8. Fortunately, the photocatalytic activity is capable of being retained for at least 5 times. Subsequently, the zinc content in the catalyst was measured by ICP. The results show that the zinc content decreases from 3 wt % to 2.8 wt %, which should be the reason for the decrease in c photocatalytic performance after five cycles.

**Figure 9:**
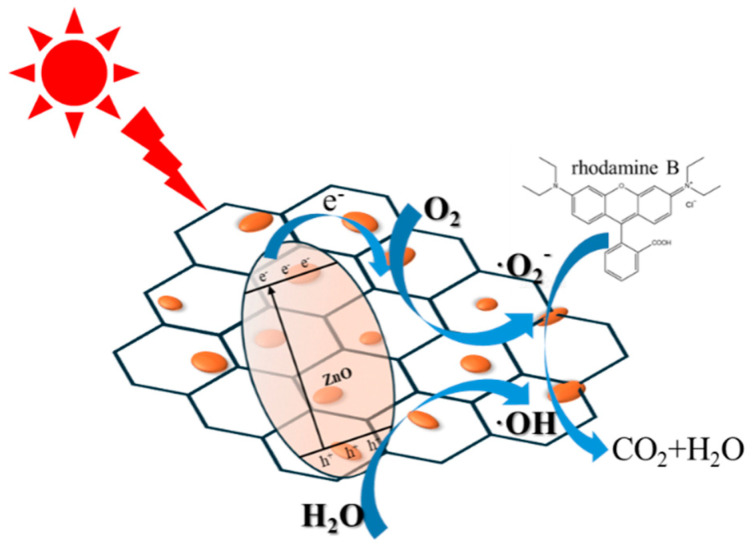
Schematic illustration of a possible RhB photodegradation process over ZGO-x.

Based on the above material characterization and performance test results, a preliminary mechanism for the enhancement in RhB photodegradation efficiency by ZGO-x is proposed, as shown in Figure 9. When the energy of the incident photon is equal to or larger than the energy gap of the composite material, ZGO-x can capture the photon, and the electrons (e^−^) in the valence band of ZnO will become unstable after absorbing the energy of the photon, and some of the electrons will cross the forbidden band and jump to the r-GO, which will capture the adsorbed oxygen on the surface and reduce it to O^2-^. At the same time, the same number of holes (h^+^) are left in the valence band to oxidize the water molecules and OH- ions adsorbed on the surface of ZnO into hydroxyl radicals with stronger activity and oxidizing ability. Ultimately, RhB is oxidized and decomposed into CO_2_, H_2_O, and other small molecules. Thanks to the good electrical conductivity of r-GO, these photo-induced electrons can continuously migrate to the r-GO sheet which is in close contact with the ZnO surface, thus realizing the effective separation of photogenerated carriers.

## 3. Experiment

### 3.1. Synthesis of r-GO

The r-GO was prepared by the redox method. A total of 1.5 g of graphite powder was mixed with 180 mL of concentrated sulfuric acid and 20 mL of concentrated phosphoric acid and stirred for 5 min. Subsequently, 9 g of potassium permanganate was slowly added, and the water bath was heated up to 35 °C with continued stirring for 60 min. The mixed solution was then heated to 50 °C and stirred for 12 h. After the solution was cooled to room temperature, a certain amount of H_2_O_2_ was added until the color of the mixture changed from purple-black to brown-yellow. Finally, the brownish-yellow solution was filtered, washed, and dried.

### 3.2. Synthesis of ZnO Nanoparticles/r-GO

**Figure 10:**
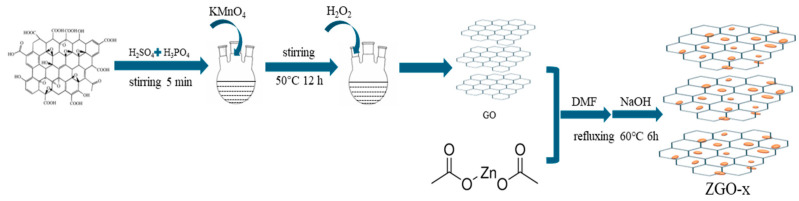
Synthesis steps of ZGO-x photocatalyst.

A total of 62.5 mL of 0.02 mol/L zinc acetate/methanol solution was mixed with r-GO. Subsequently, 1 mL of N,N dimethylamide (DMF) was added and condensed under reflux at 60 °C. The reaction was carried out for 6 h with the addition of 25 mL of 0.1 mol/L NaOH solution dropwise. The resulting samples were collected by centrifugation and subsequently washed three times using water and ethanol. The resulted final catalysts with increased Zn content were labeled as ZGO-x (x = 2.5, 3, and 3.5), and their theoretical mass ratios of Zn/r-Go are 2.5:1, 3:1, and 3.5:1 (Figure 10).

### 3.3. Catalyst Characterization

X-ray diffraction (XRD) patterns were recorded on a Rigaku SmartLab 9 kW diffractometer with Cu-Kα radiation (λ = 0.15418 nm) in the 10–85° range. N_2_ adsorption–desorption isotherms were obtained using a micromeritics ASAP-2460 instrument in static mode, and all samples were degassed at 350 °C for 3 h. The specific surface area was calculated by the Brunauer–Emmett–Teller (BET) equation, and the pore volume and average pore diameter were calculated based on the Barrett–Joyner–Halenda (BJH) method. X-ray photoelectron spectroscopy (XPS) measurements were performed on a Thermo Fisher Scientific EscaLab 250Xi instrument. The peak positions were corrected by using the containment carbon (C 1s peak = 284.8 eV).

### 3.4. Catalytic Activity Measurement

The ZGO-x series catalysts were reacted at room temperature (25 ± 2 °C). A total of 25 mg of ZGO-x catalyst was added to 50 mL of aqueous MO solution and adsorbed for 30 min at room temperature and then exposed under a xenon lamp at a light intensity of approximately 413 mW/cm^2^. A total of 3 mL of supernatant was removed at regular intervals, filtered through a 0.45 mm Millipore filter, and analyzed by a UV-vis spectrophotometer. The working electrode was prepared by dispersing 10 mg of ZGO-x in CH_3_CH_2_OH and ultrasounding for 10 min. The slurry was subsequently impregnated onto the surface of a 1 cm × 3 cm fluorotin oxide (FTO) glass substrate, which was allowed to dry overnight at room temperature. The separation efficiency of photogenerated electron–hole pairs was recorded on a CHI660D electrochemical workstation by using a standard three-electrode system. A saturated sodium sulfate solution was used as an electrolyte, graphite and Ag/AgCl electrodes were used as the counter and reference electrodes, and the sample-derived electrode was used as the working electrode. A 300 W Xe lamp (PLS-SXE300, Perfectlight Corporation, Beijing, China) with 100 mW/cm^2^ UV-Vis intensity was used as the light source.

## 4. Conclusions

In summary, we generated ZnO/r-GO composite catalysts by growing ZnO on the r-GO surface via a hydrothermal method. A series of characterizations showed that the composites retained the morphology and crystallinity of the original ZnO and exhibited strong stability and reliability. The ZGO-3 sample presented the highest degradation efficiency for RhB, which was almost 100% degraded after 150 min. The improved catalytic ability of ZnO/r-GO photocatalysts is due to the formation of a tight association between ZnO and r-GO sheets. In addition, the ZnO/rGO composites promote the reduction in the recombination of photogenerated e-h pairs. This study provides new perspectives on the formation of ZnO/r-GO composites for environmental applications.

## Figures and Tables

**Table 1:** Physical properties and parameters of ZnO and ZGO-3.

Samples	S_BET_/(m^2^/g)	V_a_/(cm^3^/g)	Band Gap (eV)
ZnO	8.1486	0.034482	3.172
ZGO-3	31.5425	0.165617	2.953

## Data Availability

Data are contained within the article.
